# Molecular Approach to Uterine Leiomyosarcoma: LMP2-Deficient Mice as an Animal Model of Spontaneous Uterine Leiomyosarcoma

**DOI:** 10.1155/2011/476498

**Published:** 2011-03-08

**Authors:** Takuma Hayashi, Akiko Horiuchi, Kenji Sano, Nobuyoshi Hiraoka, Yae Kanai, Tanri Shiozawa, Susumu Tonegawa, Ikuo Konishi

**Affiliations:** ^1^Department of Immunology and Infectious Disease, Shinshu University Graduate School of Medicine, 3-1-1, Asahi Matsumoto, Nagano 390-8621, Japan; ^2^Promoting Business Using Advanced Technology, Japan Science and Technology Agency (JST), Saitama 332-0012, Japan; ^3^Department of Obstetrics and Gynecology, Shinshu University School of Medicine, Nagano 390-8621, Japan; ^4^Department of Laboratory Medicine, Shinshu University Hospital, Nagano 390-8621, Japan; ^5^Pathology Division, National Cancer Center Research Institute, Tokyo 104-0045, Japan; ^6^Picower Institute and Department of Biology, Massachusetts Institute of Technology, Cambridge, MA 02139-4307, USA; ^7^Department of Gynecology and Obstetrics, Kyoto University Graduate School of Medicine, Kyoto 606-8501, Japan

## Abstract

Uterine leiomyosarcoma (LMS) develops more often in the muscle tissue layer of the uterine body than in the uterine cervix. The development of gynecologic tumors is often correlated with female hormone secretion; however, the development of uterine LMS is not substantially correlated with hormonal conditions, and the risk factors are not yet known. Importantly, a diagnostic-biomarker which distinguishes malignant LMS from benign tumor leiomyoma (LMA) is yet to be established. Accordingly, it is necessary to analyze risk factors associated with uterine LMS, in order to establish a treatment method. LMP2-deficient mice spontaneously develop uterine LMS, with a disease prevalence of ~40% by 14 months of age. We found LMP2 expression to be absent in human LMS, but present in human LMA. Therefore, defective LMP2 expression may be one of the risk factors for LMS. LMP2 is a potential diagnostic-biomarker for uterine LMS, and may be targeted-molecule for a new therapeutic approach.

## 1. Introduction

The uterus is the female reproductive organ, located at the center of the pelvis between the left and right ovaries. The uterus, the organ in which the embryo grows, is composed of three layers, the uterine endometrium which serves as a bed for the embryo; the myometrium of the wall which protects the embryo; and a serous membrane enveloping the uterus. The myometrium is composed of smooth muscle. In general, the term uterine tumor refers to an epithelial malignant tumor of the uterus, which is roughly classified as a tumor of the uterine cervix or the uterine body. Because of the prevalence of screening, uterine cervix cancer is decreasing in incidence, and usually detected at a very early stage, including stage 0. In contrast, cancer of the uterine body is increasing in incidence, and rarely detected at the initial stages. While most tumors of the uterine body are adenocarcinomas (derived from the subintimal gland), tumors of the uterine cervix are classified into squamous cancer and adenocarcinoma. The myometrium is composed of smooth muscle. Smooth muscle tumors (SMTs) which develop in the myometrium have been traditionally divided into benign LMA and malignant LMS based on cytological atypia, mitotic activity and other criteria. Uterine LMS, one of the most common neoplasms of the female genital tract, is relatively rare, having an estimated annual incidence of 0.64 per 100,000 women [1]. Uterine LMS accounts for 2% to 5% of tumors of the uterine body and develops more often in the muscle layer of the uterine body than in the uterine cervix [2, 3]. Distinguishing uterine LMA from uterine LMS is very difficult, and a diagnosis generally requires surgery and cytoscopy.

 The cause of tumors of the uterine cervix has been found to be the human papilloma virus, in combination with other factors. An infection is established by sexual activity. In contrast, a main factor in the development of tumors in the uterine body is the hormonal environment. Patients with uterine body tumors often are unmarried, have never been pregnant, and are taking a hormonal agent. High estrogen levels are considered to significantly influence the development of such tumors. The mechanisms by which uterine LMA and LMS develop are not yet known, though tumor cells that have developed in the myometrium for some reason gradually become larger due to the influence of the female hormone, estrogen, and generate tumors. However, no correlation between the development of uterine LMS and hormonal conditions, and no obvious risk factors have been found. The prognosis of uterine LMS is not good, and the five-year survival rate is approximately 35%, although the five-year survival rate depends on disease stage [2, 3]. It is worth noting that, when adjusted for stage and mitotic count, LMS has a significantly worse prognosis than carcinosarcoma [4]. As uterine LMS is resistant to chemotherapy and radiotherapy, and thus surgical intervention is virtually the only means of treatment [5–7], developing an efficient adjuvant therapy is expected to improve the prognosis of the disease. Although cases accompanied by hypocalcaemia or eosinophilia have been reported, neither clinical abnormality is an initial risk factor for uterine LMS. The identification of a risk factor associated with the development of uterine LMS would significantly contribute to the development of preventive and therapeutic treatments.

## 2. Biological Roles of the Immunoproteasome

**Figure 1:**
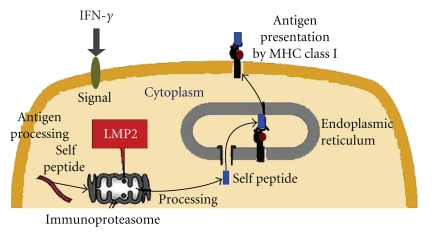
Mediation of the proteasomal degradation pathway to antigen presentation by MHC class I. The immunoproteasomal degradation pathway is essential for antigen presentation by MHC class I. Defecive LMP2 expression results in tissue- and substrate-dependent abnormalities of immunoproteasomal functions. Therefore an impaired proteasome may promote the initial development of disease including tumorigenesis.

 When tissue or an organ is transplanted, the graft is often lost due to an acute rejection caused by the host immune system. This is because the cell surface antigens presented by the major histocompatibility complex (MHC) are intrinsic to an individual and so differ between the donor and recipient. The immunological self markers on cell surfaces are the most important immune system for higher vertebrates such as mammals, protecting the self from invaders. Cytoplasmic proteins are mostly degraded by a protease complex, which has many substrates consisting of twenty-eight 20 to 30-kDa subunits, referred to as the immunoproteasome [8]. The proteasomal degradation pathway is essential for many cellular processes, including the cell cycle, the regulation of gene expression, and others. The proteasomal degradation pathway is also essential for the production of peptide antigens which are presented by MHC class I. That is, the immunoproteasome plays a key role in the presentation of immunological self markers on the cell surface by MHC (Figure 1) [8]. Interferon-*γ* (IFN-)*γ*) is a critical inducer of the immunoproteasome's expression in immune systems [9]. Recent findings have verified that IFN-*γ* prevents primary tumor development, thereby showing a tumor suppressor role in the immune response [10, 11]. IFN-*γ* upregulates the expression of large numbers of responsive genes, also, expression of the immunoproteasome's subunits, that is, low-molecular mass polypeptide (LMP) 2, LMP7, and LMP10, is markedly induced by IFN-*γ* signaling [9, 12]. The IFN-*γ*-inducible proteasomal function plays a key role in MHC class I-mediated tumor rejection [11, 13]. Further, a molecular approach to studying the correlation of IFN-*γ* with tumor cell growth has drawn attention. A deficiency of IFN-*γ* apparently does not hamper the generation of CTL [10, 11]. Recent reports have demonstrated the multifunctional deficiencies of components of the MHC class I antigen-presentation pathway including LMP2 and TAP-1 in tumor cells [11, 13]. A possible role for the IFN-*γ*-responsive gene *TAP-1 *in tumor recognition was reported [11]. Here we identify LMP2, a single IFN-*γ*-responsive gene product, as obligatory for tumor surveillance [12] and demonstrate a tissue-specific role for LMP2 in protection from spontaneous neoplasms of the uterus.

## 3. Development of Malignant Uterine Tumor in LMP2-Deficient Mice

Malignant tumors originate from a single cancerous cell and develop as a result of unlimited cell proliferation. Malignant tumor cells have properties that are biologically different from those of normal cells. Thus, the host immune system should be able to distinguish malignant tumor cells from corresponding normal cells. That is, malignant tumor cells present intrinsic antigens (i.e., tumor-cell-specific antigens that can be the targets of immune responses are referred to as tumor-antigens (TA)) on the cell surface with the aid of MHC. In many cases, however, almost no reaction by the immune system is observed. Also, the incidence of major tumors is not very different between immunodeficient (i.e., lymphocyte-deficient) mice and control mice having normal immune systems. Specifically, tumor cells can avoid the immune monitoring system via several means [14, 15]. Naturally occurring tumor cells seem to have lost the expression of peptide antigens, TA, or cell adhesion factors intrinsic to tumors. Tumor cells may avoid the host immune reaction due to the absence of MHC expression, although no such mechanism has yet been elucidated. However, it is important to demonstrate how tumor cells evade immune-responses, in order to prevent the development of tumors.

**Figure 2:**
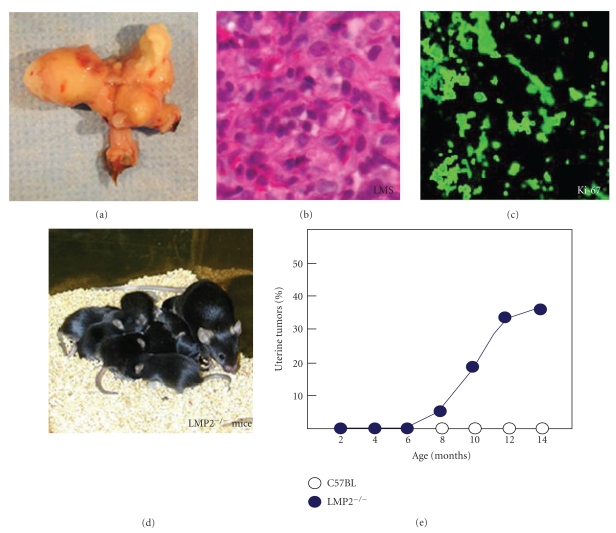
Histological findings of uterine leiomyosarcoma in LMP2-deficient mice. Histological findings of uterine LMS in LMP2-deficient mice ((a) to (c)). Among the histological findings of uterine LMS in LMP2-deficient mice, a cytoskeleton, which is characteristic of uterine LMS, is observed. ((b) and (c) magnification x400) Panel (e), in LMP2-deficient females, uterine LMS is observed at 6 months of age. The incidence at age 14 months is as high as 40% (e). The curve indicating the incidence of mouse uterine LMS is very similar to that indicating the incidence of human uterine LMS, which is observed after menopause. In mice with tumors of the uterus, significant weight loss is observed. Thus, a tumor that develops in the uterus is diagnosed as malignant, that is, uterine LMS.

The genes encoding LMP2, LMP7, TAP1, and TAP2, are located in region H-2 which encodes the murine MHC molecule. LMP2-deficient mice show tissue- and substrate-dependent abnormalities in the biological functions of the immunoproteasome, and impaired functioning of the immunoproteasome in the spleen or hepatic cells [16]. Further, LMP2-deficient mice do not show normal immune responses to virus-infected cells, and such immunopathy is known to result from a failure in the presentation of peptide antigens on the cell surface by MHC [16]. We found that uterine LMS occurred in female LMP2-deficient mice at age 6 months or older, and the incidence at 14 months of age was about 40% [17] (Figure 2). The curve indicating the incidence in mice is very similar to that indicating the incidence of human uterine LMS, which occurs after menopause. Histological examinations of LMP2-lacking uterine tumors revealed characteristic abnormalities of LMS [17]. The tumors lacked lymphoid infiltrates, a sign of immune recognition, and consisted of uniform elongated smooth muscle cells arranged into bundles. The nuclei of the tumor cells varied in size and shape; furthermore, mitosis was frequent, in contrast, the uterine smooth muscle cells of C57BL/6 mice were normal in appearance [17]. Whereas relatively few Ki-67-positive cells, the proliferating cells of solid tumors, were observed in the basal cell layer of the normal uterine smooth muscle, most of the basal cells vividly expressed Ki-67 in LMP2-deficient mice [17]. This immunohistochemical (IHC) staining indicates abnormal proliferation of the LMP2-lacking cells in the basal layer [17] (Figure 2). LMP2-deficient mice that have developed uterine LMS undergo considerable weight loss, and then die by 14 months of age [17]. The LMP2-deficient mice also exhibit skeletal muscle metastasis from uterine LMS. Therefore it is like LMP2-deficient mice with uterine LMS have died of mass effect and metastasis. In general, it is not easy to distinguish uterine LMA from LMS. However, in mice, because of such characteristic pathological findings, significant weight loss, and exhibition of skeletal muscle metastasis, a tumor that develops in the uterus of an LMP2-deficient mouse can be considered malignant, that is, a uterine LMS. 

If the *TP53* gene is damaged, tumor suppression is severely reduced. People who inherit only one functional copy of the *TP53* gene will most likely develop tumors in early adulthood, a disease known as Li-Fraumeni syndrome. More than 50 percent of human tumors contain a mutation or deletion of the *TP53* gene [18]. To increase tumor incidence and better assess the role of systemic expression of TP53 in responses to initiation of uterine LMS tumorigenesis, LMP2-deficient mice were bred with TP53-deficient mice to create *Lmp*2^−/−^ *Tp*53^−/−^ double knockout mice. Uterine LMS incidence and death rates were similar in *Lmp*2^−/−^ *Tp*53^−/−^ mice and closely matched control *Lmp*2^−/−^ *Tp*53^+/+^ mice. The correlation of defective TP53 function with uterine LMS tumorigenesis is not clearly understood.

## 4. Inactivation of the IRF-1 Tumor Suppressor Gene in LMP2-Deficient Mice

Uterine LMS was demonstrated to spontaneously develop in 6-month-old LMP2-deficient mice at high frequency. The expression of LMP2 was significantly induced by IFN-*γ* as was the expression of other subunits [9, 12]. Accordingly, the expression of cell-cycle regulators that are regulated by the IFN-*γ* signal cascade or immunoproteasome activity was examined. Signal transducer and activator of transcription (STAT) 1, having been activated by IFN-*γ*, significantly induced expression of tumor suppressors such as interferon regulatory factor 1 (IRF1) [19, 20]. IRF1 as a transcriptional regulator significantly regulates LMP2 expression [19, 20]. It was examined whether the IFN-*γ* signal cascade induces the expression of each subunit of the immunoproteasome and IFR1 and IRF2 in LMP2-deficient mice and the parental strain, C57BL/6. No significant difference was observed in the expression of STAT1 and the subunits LMP7, LMP10, CP9, and IRF2. Also, IFN-*γ*-induced phosphorylation of STAT1 would not be influenced by a lack of LMP2. However, the expression of IRF1 was significantly reduced in splenocytes derived from mice lacking LMP2 in comparison with wild-type mice. IRF1 expression in LMP2-deficient splenocytes was not induced by the IFN-*γ* signal cascade. In addition, wild type-mouse embryonic fibroblasts (MEFs) that had been treated with the proteasome inhibitor MG-132 exhibited a loss of IFN-*γ*-inducibility, reproducing a phenotype of the LMP2-deficient mouse. Accordingly, the transcription of *Irf1* mRNA depends on the immunoproteasome's function and is considered to involve the formation of a STAT1 homodimer. Recent reports suggest that proteasomal function contributes to mRNA transcriptional activation [21, 22]. 

**Figure 3:**
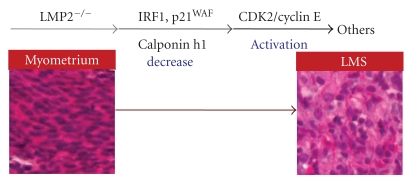
Model of the mechanism for development of uterine leiomyosarcoma. In LMP2-deficient cells, levels of the antioncogenic factor IRF-1, p21^WAF^ are significantly reduced. Reduced expression of the calponin h1 transcript, which contributes to cell proliferation and tumorigenesis in uterine smooth muscle cells, is detected in uterine LMS tissues. Cell cycle regulatory factors, CDK2/Cyclin E, are markedly activated. The inactivation of such antioncogenic factors is considered to transform LMP2-deficient cells into malignant tumor cells.

Primary cultured tumor cells (LMP2-UC) were established from the uterine LMS of LMP2-deficient mice, and then IRF1-overexpressing tumor cells (LMP2-UC-IRF1) were further established by genetic engineering. The LMP2-UC-IRF1 cells were intracutaneously transplanted into immunodeficient mice (BALB/c nu/nu), and significant inhibiting effects of IRF1 on tumor cell proliferation were observed [20, 23]. Thus, a lowered level of IRF1 resulting from a deficiency in LMP2 seemed to be a risk factor for uterine LMS in mice. The effects of IRF1 on tumor cell proliferation are achieved through the expression of p21^WAF^ cell-cycle inhibitors (inhibiting transition from the G1 to S stage) [24]. Whether or not p21^WAF^ expression or activation is affected in LMP2-deficient mice should be examined further. The tumor suppressor, retinoblastoma (Rb) is phosphorylated by a complex of Cyclin E/Cyclin dependent kinase 2 (CDK2) and then inactivated [25]. Also, the activity of CDK2 is negatively regulated via degradation of Cyclin E by the 26S proteasome [26, 27]. A significant level of phosphorylated-Rb is observed in MEFs-lacking LMP2, and the activity of CDK2 for phosphorylation is determined to be stronger than that in normal MEFs. However research overall, including experiments with gene-deficient mouse models and clinical studies, suggests that defective Rb expression does not take part in the onset of uterine LMS [28–30]. In the case of uterine LMS in LMP2-deficient mice, defective IRF1 is considered to be involved in cellular transformation and cell proliferation (Figure 3).

## 5. Perspectives

Uterine LMS mainly develops in the uterine smooth muscle or endometrial stroma, and menstrual anomalies, such as hypermenorrhea and prolonged menstruation, and symptoms such as abnormal hemorrhage, hypogastric pain, lumbar pain, and abdominal strains, are observed [4]. In the case of gynecological cancers, such as breast cancer, a female hormonal imbalance is often a risk factor for developing tumors. As in the case of uterine LMA, however, a correlation between the development of uterine LMS, the female hormone, and hormone receptors has yet to be elucidated [31, 32]. A recent report showed the expression of *Lmp2* mRNA and protein in luminal and glandular epitheliua, placenta villi, trophoblastic shells, and arterial endothelial cells [33]. These results implicate LMP2 in the invasion of placental villi, degradation of the extracellular matrix, immune tolerance, glandular secretion, and angiogenesis [33]. The present study should help to elucidate the regulatory role of the ubiquitin-proteasome pathway in the implantation of embryos. Unfortunately, it is unclear whether defective LMP2 expression is involved in the onset of uterine LMS. Uterine LMS often seems to develop in individuals exposed to radiation in the pelvis. Risk factors for its development, however, have not been identified because of the absence of a suitable animal model. The LMP2-deficient mouse was the first animal model of spontaneous uterine LMS to be established [17]. Defective LMP2 expression may be one of the causes of uterine LMS [20]. To demonstrate whether LMP2 is a potential biomarker for distinguishing LMS from LMA, we are investigating the reliability and characteristics of LMP2 as a diagnostic indicator with several clinical research facilities. The clinical research is yet to be concluded, and large-scale clinical studies need to be performed. In some cases, uterine LMA may become malignant and develop into uterine LMS. Accordingly, the correlation between the inactivation of LMP2 and the development of uterine LMA needs to be examined. Although LMS usually lacks lymphoid infiltrates recognizable on routine histological staining, further histological examination revealed a few infiltrating CD56^+^ natural killer cells in human uterine LMS tissues. Definitive histological studies must be performed, including the gene-expression profiling of several known pro-oncogenic factors as well as factors such as brain-specific polypeptide PEP-19 and a transmembrane tyrosine kinase receptor, C-kit [34–36]. The reduced expression of calponin h1 transcripts was reported to be associated with uterine LMS, and calponon h1 might function as a tumor suppressor in uterine LMS [37, 38]. A recent study showed that re-expression of human calponin h1 suppressed cell proliferation and tumorigenesis in uterine LMS cells [38]. Since no spontaneous development of uterine LMS is observed in IRF1-, calponin h1-deficient mice or heterozygous Rb mice, the lack of LMP2 is largely associated with the expression of other known or unknown cell-cycle regulatory factors. Further research is required to demonstrate the correlative functions of LMP2 and other antioncogenic factors with calponin h1 in the tumorigenesis of uterine LMS. Clarification of the correlation between these factors and the development of uterine LMS and the identification of specific risk factors may lead to the development of new treatments for the disease. Uterine LMS is refractory to chemotherapy and has a poor prognosis. The molecular biological and cytological information obtained from LMP2-deficient mice will contribute remarkably to the development of preventive methods, a potential diagnostic-biomarker, and new therapeutic approaches against uterine LMS.
